# Clinical and radiographic success of pulpotomy and pulpectomy in primary and permanent teeth: a Systematic Review and Meta-Analysis

**DOI:** 10.4317/jced.61346

**Published:** 2024-09-01

**Authors:** Melissa Cavalcante Bastos, Fontenele Gilsara Araújo Albuquerque, Edson Luiz Cetira Filho, Paulo Goberlânio de Barros Silva, Juliana Paiva Marques Lima Rolim

**Affiliations:** 1 Dentistry, Christus University Center (Unichristus), Fortaleza - Ceará, Brazil

## Abstract

**Background:**

This systematic review evaluated the long-term clinical effectiveness of Biodentine in vital pulp therapy procedures.

**Material and Methods:**

Two independent reviewers searched the PubMed, Scopus, Web of Science, Cochrane, LILACS, and DOSS databases for articles published until August 2023. Review Manager and GRADEpro software were used for the analysis, and the Revman5.3 program was used for the meta-analysis. Initially, 297 publications were found, of which 12 studies, including 1385 clinical evaluations and 881 radiographic evaluations, were considered for qualitative and quantitative analysis.

**Results:**

Regarding the therapeutic indication, Biodentine presented a clinical success rate of: 96.38% for primary teeth submitted to pulpotomy, in a follow-up of 3-24 months, 97.18% for permanent teeth submitted to direct pulp capping in a follow-up of 3-54 months and 99.24% for primary teeth submitted to indirect pulp capping at a follow-up of 3-12 months. In radiographic analyses, a success rate of: 89.82% was observed for primary teeth submitted to pulpotomy at a follow-up of 3-24 months and for permanent teeth submitted to indirect pulp capping at a follow-up of 3-12 months.

**Conclusions:**

Biodentine is a reliable material for applications similar to that of mineral trioxide aggregate, with high long-term clinical and radiographic success rates, in primary and permanent teeth, regardless of the therapeutic indication. The efficacy and benefits of Biodentine, make it a promising endodontic material.

** Key words:**Systematic review, Dental materials, Pulpotomy.

## Introduction

With the advent of bioactive dental materials, there has been a broad interest in the preservation and regeneration of pulp tissue debris (1). Vital pulp therapy is a current field in endodontics that involves regenerative procedures of the dentin-pulpal complex, including indirect pulp capping, direct pulp capping, and pulpotomy (2). These therapies that aims to treat teeth with compromised dental pulp without the full removal of all healthy pulp tissue, as an alternative to endodontic treatment (3).

In addition to the type of exposure and procedure adopted, the material used strongly influences the treatment outcome (4). Therefore, an ideal material that is suiTable for these therapies must possess physical characteristics, such as sealing, dimensional and color stability, radiopacity, insolubility in contact with fluids, fluidity, and easy insertion. In addition, the chemical and biological properties should include an alkaline pH, calcium ion release, bioactivity, biocompatibility, and cell adhesion (5).

Materials containing calcium silicate (CS) are commonly used in vital pulp therapy owing to their biocompatibility and bioactive capacity (4). On the other hand, mineral trioxide aggregate (MTA) is considered the gold standard material, as it exhibits antibacterial activity and high sealability, stimulates hard tissue production, and is biocompatible (6). However, MTA has certain limitations, such as tooth discoloration. In order to suppress this effect, formulations containing lower amounts of iron, aluminum, and magnesium, such as white MTA, have been suggested (7). However, long-term studies have shown that these modifications are not sufficient to overcome this problem (8). Moreover, the long setting time (9), handling difficulties, and high cost (4) are still persistent limitations of this cement.

As a result, new materials and modifications of current formulas have been developed, including tricalcium silicate-based cements (5), such as Biodentine, which has been recognized as a promising material (10) and suggested to be superior to other silicate calcium based cements (4). To use Biodentine, a powder and liquid are combined to form a composite. The powder consists mainly of tricalcium silicate, dicalcium silicate, calcium carbonate, and zirconium dioxide, while the aqueous component contains water, calcium chloride, which accelerates its hardening, and a modified polycarboxylate, which serves as a superplasticizer.

The main development goals of Biodentine are to combine the bioactivity and high biocompatibility offered by calcium silicates with enhanced, fast setting (hardening) properties (1,4), higher strength (1,6), absence of tooth discoloration (9), and ease of handling (4). Thus, it has the same clinical applications as MTA but with superior mechanical and physicochemical properties (9). However, the literature still lacks long-term follow-ups that provide a definitive consensus on its effectiveness (10).

This systematic review aims to answer the following question: “What is the long-term clinical effectiveness of Biodentine in deep caries lesions?” This review will provide a scientific, evidence-based decision-making process for clinicians and health care professionals, through a longitudinal follow-up analyzing the clinical and radiographic success rates of Biodentine used in vital pulp therapy procedures.

## Material and Methods

The Preferred Reporting Items for Systematic Reviews and Meta-Analyses (PRISMA, http://www.prisma-statement.org/statement.htm) was followed for this systematic literature review. In addition, this study was registered in the International Prospective Register of Systematic Reviews (PROSPERO) (identification number: CRD42020212276).

-Focus Question

To direct the systematic review, the following PICOS question was formulated: “What is the long-term clinical effectiveness of Biodentine in deep caries lesions?”

Population (P): humans with deep caries (*in vivo*). Intervention (I): Application of Biodentine for indirect; pulp capping, direct pulp capping, and pulpotomy in primary and permanent teeth; Comparison (C): comparison between the control groups; Outcome (O): clinical effectiveness of Biodentine, success and/or failure rate; Studies (S): randomized clinical trials.

-Inclusion and Exclusion Criteria

Two authors independently evaluated the articles identified by the search strategy according to the following inclusion criteria: randomized clinical studies, the applicability of Biodentine in indirect pulp capping or direct pulp capping or pulpotomy, and follow-up of treatment effectiveness with success and/or failure rates on a longitudinal basis, regardless of the language of publication.

Duplicate studies, without postoperative follow-up, without a control group, or those that used Biodentine associated with other therapies were excluded. In addition, *in vitro* studies, animal experimentation, case reports or series, chart analyses, publication of protocols, and literature reviews were also eliminated to direct the review to clinical studies.

-Search Strategy

Based on the PICO question, the searches were conducted in six electronic databases: PubMed, Scopus, Web of Science, Cochrane, LILACS, and DOSS, covering publications from August 2010 to August 2023, including reports published in English and Portuguese.

The search strategies developed in each database (ANNEX 1) used the various groupings of the following terminologies: (“Dental Pulp Capping” OR “Pulp Capping, Dental” OR “Pulp Capping” “Capping, Dental Pulp” OR “Cappings, Dental Pulp” OR “Dental Pulp Cappings” OR “Pulp Cappings, Dental” OR Pulpectomy OR Pulpectomies OR “Pulp Capping and Pulpectomy Agents” OR “Pulp Capping Agents” OR “Agent, Pulp Capping” OR “Capping Agents, Pulp” OR “Pulp Capping Agent” OR “Pulpectomy Agents” OR “Agent, Pulpectomy” OR “Agents, Pulpectomy” OR “Pulpectomy Agent”) AND (“tricalcium silicate” OR biodentine OR “tricalcium silicon pentaoxide” OR Ca3SiO5) AND (clinical OR trial OR “clinical trial” OR “clinical trials” OR “random*” OR “random allocation” OR “therapeutic use”).

-Data Collection Quality Assessment

Two authors independently selected the studies (JPMLR and MCB) to minimize inconsistencies and selection bias. Disagreements between the selected studies were resolved through discussion, with no need for a third reviewer to intervene. In the initial data collection phase, the titles of the identified studies were analyzed. The next phase involved reading the abstracts. The selected reports were then read in their entirety. The last stage consisted of selecting the studies for qualitative analysis.

The evaluation of methodological quality was performed using parameters observed and adapted from the literature as follows: sample calculation (yes=1; no=0); randomization (yes=1; no=0); presence of a control group (yes=1; no=0); blinding (yes=1; no=0); methodological detailing (yes=1; no= 0), and calibration (yes or single operator=1, no=0), such that the maximum score was 6 points (11,12) ( Table 1).

-Risk of bias in individual studies

Risk of bias will be assessed independently by two review authors (J.P.M.L.R. and M.C.B.). It will be considered the Joanna Briggs Institute critical appraisal checklist for randomized controlled trials, as follows: 1) Was true randomization used for assignment of participants to treatment groups?; 2) Was allocation to treatment groups concealed?; 3) Were treatment groups similar at the baseline?; 4) Were participants blind to treatment assignment?; 5) Were those delivering treatment blind to treatment assignment?; 6) Were outcomes assessors blind to treatment assignment?; 7) Were treatment groups treated identically other than the intervention of interest?; 8) Was follow up complete and if not, were differences between groups in terms of their follow up adequately described and analyzed?; 9) Were participants analyzed in the groups to which they were randomized?; 10) Were outcomes measured in the same way for treatment groups?; 11) Were outcomes measured in a reliable way?; 12) Was appropriate statistical analysis used?; 13) Was the trial design appropriate, and any deviations from the standard RCT design (individual randomization, parallel groups) accounted for in the conduct and analysis of the trial?

-Assessing the Certainty of Scientific Evidence

The assessment of the certainty of scientific evidence was performed using the online software GRADEpro, where the main study results were analyzed for: 1) number of included studies, 2) study design, 3) risk of bias, 4) inconsistency, 5) indirect evidence, 6) imprecision, 7) number of patients, and 8) effect, thus obtaining the certainty of the evidence and its significance.

-Meta-analysis

Data were imported into an Excel spreadsheet (Microsoft Corporation) to obtain the relative and absolute frequencies, and the meta-analysis prevalence was calculated using MedCalc 18.2.1 software (MedCalc®) with a 95% confidence interval (CI) and random-effect model. An I-squared (I²) test was used for the heterogeneity analysis. Additionally, subgroup analyses by the time of evaluation or type of treatment (direct pulp capping, indirect pulp capping, or pulpotomy) were conducted.

## Results

-Study Selection

According to the search protocol, a total of 297 reports were found in the following databases: MedLine/PubMed (n=71), Scopus (n=83), Web of Science (n=58), Cochrane (n=34), LILACS (n=1), and DOSS (n=50). After eliminating duplicates, 158 studies remained in the title and abstract reading phase. In this phase, 128 papers were excluded, including 50 *in vitro* studies, 30 literature reviews, 19 case reports, 14 animal experiments, 14 protocol publications, and one medical record analysis.

Seven articles were excluded in the complete reading stage: four for using Biodentine treatment associated with external factors, such as the use of laser (21-24), one for presenting analysis only of restorative durability (25), one for not presenting longitudinal results, containing only postoperative analysis (26), and one for containing partial results (preliminary) of an article already included in the review (27). Thus, 23 articles were selected for the eligibility analysis, 11 were eliminated, five did not contain a control group, and six were of low methodological quality, lacking sample calculation and methodological details. A total of 12 studies were selected for qualitative analysis: Awawdeh *et al*. (9); Bani *et al*. (13); Brizuela *et al*. (14); Carti and Oznurhan (6); Caruso *et al*. (15); Çelik *et al*. (4); Cuadros-Fernández *et al*. (1); Garrocho-Rangel *et al*. (16); Hashem *et al*. (17); Katge and Patil (18); Parinyaprom *et al*. (19); Rajasekharan *et al*. (20).

**Figure 1:**
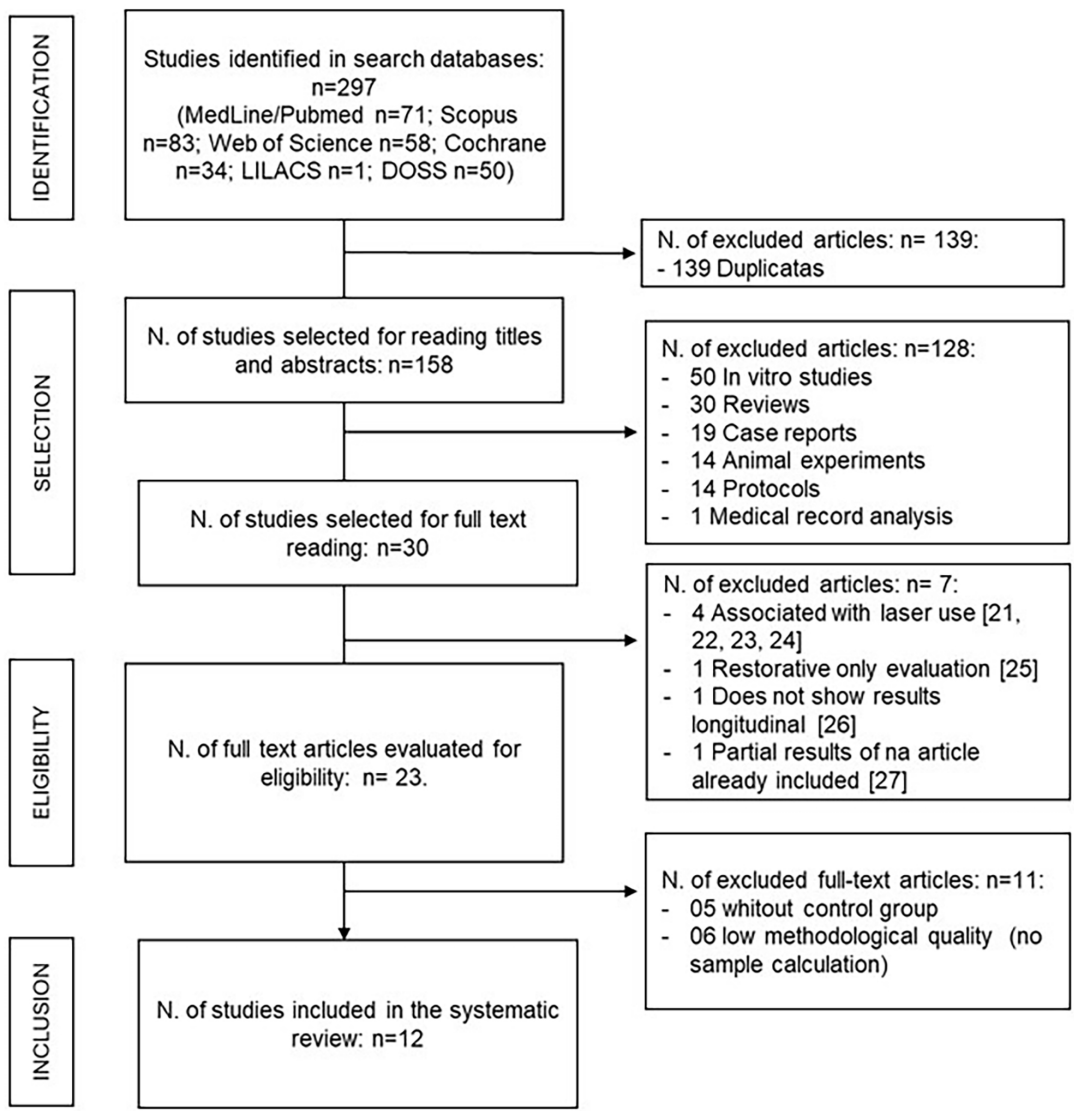
Flowchart (PRISMA Model): Flow diagram of the study identification, selection, eligibility and inclusion of the studies.

The following flowchart describes the article search and selection steps according to the PRISMA recommendation model (Fig. 1).

-Deciduos dentition 

In the study developed by Carti and Oznurhan (6), the clinical and radiographic effectiveness of MTA and Biodentine in performing pulpotomies in 50 deciduous teeth at 3, 6, and 12 months were evaluated. The respective clinical and radiographic success rates in the groups presented were 96% and 80% for MTA and 96% and 60% for Biodentine, with no significant difference between the groups. Thus, Biodentine achieved success rates similar to those of the control and was considered safe in pulpotomies.

Cuadros-Fernández *et al*. (1) obtained similar results in their analysis. The pulpotomies were still performed in the deciduous dentition, but included a sample size of 84 molars, with a clinical and radiographic follow-up at 6 and 12 months. At 12 months, the clinical evaluation generated success rates of 92% for the MTA group and 97% for the Biodentine group, with failures observed because of gingival inflammation. Regarding the radiographic results, MTA obtained 97% success rate, as one molar presented resorption, while Biodentine presented 95%, because of one resorption and one radiolucency observed. Therefore, Biodentine showed comparable results to MTA and can be used in the pulpotomy of deciduous molars.

Çelik *et al*. (4) performed pulpotomies on 44 lower deciduous molars, following them up for a longer period of 24 months. The overall success rates obtained at the end of follow-up were 100% for the MTA group and 89.4% for the Biodentine group. Although failures were found in the pulp canal obliteration in the MTA group at 6 months, they stabilized over treatment. Thus, the longitudinal follow-up demonstrates the effectiveness of Biodentine and MTA for pulpotomy in deciduous teeth.

The clinical trial by Caruso *et al*. (15) aimed to compare the success of pulpotomies performed on 400 deciduous molars when calcium hydroxide or Biodentine was used. The combined clinical and radiographic success rates in the analyses performed at 9 and 18 months for calcium hydroxide (CH) were 85.5% and 81.5%, respectively, while Biodentine showed 94% and 89.5% success rates, respectively, with the latter showing a significantly higher efficacy. In addition, the findings showed that Biodentine has greater clinical and radiographic success after 18 months compared to that of CH; however, this new material has a higher cost and a longer setting time than that of CH.

Garrocho-Rangel *et al*. (16) compared the effects of CH and Biodentine in indirect pulpal capping. A total of 160 deciduous teeth were selected for follow-up at 1, 3, 6, and 12 months. The overall results combining clinical and radiographic rates showed success rates of 98.3% for Biodentine and 95% for CH. Therefore, both materials were suiTable for this procedure, without significant differences in their results.

Rajasekharan *et al*. (20) compared the differences among three agents, MTA, Biodentine, and Thempophore, in pulpotomies of deciduous molars. After treatment for 6, 12, and 18 months, 82 teeth were selected and followed up clinically and radiographically. After 18 months, the clinical and radiographic results were 95.24% and 94.4% for Biodentine, 100% and 90.9% for MTA, and 95.65% and 82.4% for Thempophore. Thus, there was no significant difference in treatment between the three agents; other factors analyzed, such as sex, age, operator, treated tooth, and others, did not seem to influence the efficacy of the materials.

Bani *et al*. (13) compared the effectiveness of Biodentine and MTA in a pulpotomy procedure with a follow-up of 24 months in 64 deciduous teeth. At the end of clinical follow-up, the success rates were equivalent between Biodentine and MTA (96.8%), while Biodentine had a higher success rate of 93.6%, compared to MTA at 87.1%. The results were not significantly different during follow-up between the groups, and both were effective in this treatment.

-Permanent dention

Awawdeh *et al*. (9), aimed to compare the clinical and radiographic efficacy of Biodentine and MTA in the treatment of direct pulpal capping. For this, 68 patients with deep caries, who underwent direct pulp capping were followed up over 6 months and 1, 2, and 3 years; however, in cases where there was a failure in the procedure, such as lack of pulp tissue hemostasis, the dental elements underwent pulpotomy. MTA was found to have success rates of 93.5%, 100%, 100%, and 96%, respectively, while Biodentine achieved success rates of 93.1%, 96%, 100%, and 91.7%, respectively. Therefore, Biodentine achieved a similar efficacy rate as that of MTA in direct capping procedures as well as in pulpotomy of permanent teeth.

In addition to assessing the clinical performance of MTA and Biodentine as direct pulp capping materials, Parinyaprom *et al*. (19) analyzed the gray discoloration generated by both materials. To this end, 59 permanent teeth were evaluated at time intervals ranging from 6 to 54 months. At the mean follow-up, 18.9 ± 12.9 months, MTA was 92.6% successful, while Biodentine was 96.4% successful. Grayish colorations were observed only in MTA, with an incidence of 55%. Therefore, Biodentine showed a superior result when compared to the standard choice, MTA, in direct pulp capping treatments, and it did not change the color of the tooth.

Katge and Patil (18) studied the effectiveness of Biodentine and MTA through a split-mouth clinical trial. They analyzed 58 permanent molars undergoing direct pulp capping treatments at 6 and 12 months. The clinical and radiographic results showed 100% success for both materials during the entire follow-up period. In addition, dentin bridge formation was higher at 6 months in the MTA group and 12 months in the Biodentine group, but this was also a non-significant difference. Therefore, both materials induce remineralization in the pulpal tissue and are suiTable for direct pulpal capping treatment.

Brizuela *et al*. (14) analyzed the replacement of the gold standard material, CH, with CS materials, such as MTA and Biodentine, in direct pulp capping operations. A total of 169 teeth were selected for treatment and randomly assigned to one of the three groups, and clinical follow-up examinations were performed at 1 week, 3 months, 6 months, and 1 year. Biodentine presented a 100% success rate during the follow-up, with success rates ranging from 100% to 86.36%, and from 97.22% to 86.36% among the CH group. Although no significant differences in results were found, calcium silicate-based materials seem to be adequate substitutes for CH. Biodentine, in addition to its high success rate, was associated with certain advantages, such as easy handling, fast adjustment, and no discoloration of the dental structure.

The Hashem and colleagues (17) study aimed to evaluate Biodentine and Glass Ionomer Cement as indirect pulp capping materials at a 2-year follow-up. A total of 72 restorations were randomly performed with the materials, with clinical and radiographic follow-ups. At 24 months, Biodentine showed six teeth with loss of pulp vitality, while glass ionomer cement showed nine teeth under the same conditions. Therefore, teeth with pulpitis that require indirect pulp capping can be effectively treated with both materials.

-Meta-analysis Results.

Deciduos dentition

In the meta-analysis of clinical evaluation of primary teeth selected for pulpotomy, the survival of restorations with biodentin was 98.90% (95%CI = 93.91 to 99.84%) after three months, remaining at 98.23% (95%CI). % = 94.73 to 99.88) after six months. After 9-12 months of follow-up, survival was slightly lower than 96.50% (CI95% = 99.83 to 98.38%), remaining without significant difference after 18 months, with a survival of 95.88% (CI95% = 93.21 to 97.91%) and 24 months, with a survival of 92.82% (CI95% = 84.16 to 98.23%). In none of the periods, however, was there significant heterogeneity (I² = 0 to 9.80%), there was a significant risk of publication bias after 3 months (Egger’s test: *p*<0.001), 9-12 months (Begg’s test: *p*= 0.019) and 24 months (Egger test *p*<0.001).

In the meta-analysis of radiographic evaluation in primary teeth submitted to pulpotomy, the survival of restorations with biodentine was 86.40% (CI95% = 69.617 to 97.08%) after three months, 93.48 (CI95% = 80.86 to 99.59%) after six months. After 9-12 months of follow-up, survival was slightly less than 96.50% (CI95% = 99.83 to 98.38%), with no significant difference after 9-12 months, with a survival of 88.91% (CI95% = 79. 69 to 95.59%). The values changed little after 18 (89.92%, CI95% = 86.09 to 93.19%) and 24 months (89.09%, CI95% = 79.40 to 95.98%). Between 3 and 12 months there was significant heterogeneity (I² = 51.51 to 75.18%) but there was significant publication bias only after 24 months (Egger’s test: *p*<0.001).

Permanent dentition

In the meta-analysis of clinical evaluation of permanent teeth submitted to direct pulp capping, the survival of restorations with biodentine was 98.75% (CI95% = 96.23 to 99.91%) after 3-6 months, remaining at 97, 78% (CI95% = 93.34 to 99.85%) after 10-12 months of follow-up. The survival of these restorations was only slightly less than 94.96% (CI95% = 90.26 to 98.18%) after 18-54 months. There was no significant heterogeneity in any of the periods evaluated (I² = 0%), but there was a risk of publication bias.

Only Garrocho-Rangel *et al*., 2017 (16) clinically evaluated primary teeth after indirect pulp capping in a period of 3 to 12 months, obtaining a survival rate of 99.24% (CI95% = 97.62 to 99.96%). Only Katge and Patil., 2017 (18) radiographically evaluated permanent teeth submitted to indirect pulp capping, observing a survival rate of 98.85% (CI95% = 93.62 to 99.83%). Hashen *et al*., 2019 (17) was the only study that clinically evaluated permanent teeth submitted to indirect pulp capping in a period of 24 months, obtaining a survival rate of 77.8%.

-Result of the Certainty of Evidence Analysis

Analysis of the certainty of evidence for the clinical and radiographic outcomes of the success rate of Biodentine, regardless of treatment, resulted in a high level of certainty ( Table 2).

## Discussion

Vital pulp therapy includes a series of techniques, such as indirect pulp capping, direct pulp capping, and pulpotomy, with the main goal of maintaining the integrity and health of the pulp tissue by preserving its vitality and stimulating regeneration of the dentin-pulp complex (18). The success of this treatment is correlated to several factors, including the clinical situation, aseptic operative technique, and the biomaterials used during the procedure (28). Endodontics aims at preserving pulpal tissues, changing the focus to pulpal therapies of vital teeth, where a better long-term prognosis is observed (29).

The mechanism of action of MTA works by inducing the formation of crystalline structures through the reaction of calcium oxide with tissue fluids and CH, which interacts with CO2 from the bloodstream to form calcium carbonate. The secretion of fibronectin then initiates the formation of hard tissues. Histologically, this tissue deposition occurs through calcite granulation, which, in the presence of fibronectin provides adhesion and odontoblast-like cell differentiation and initiates the formation of the dentin barrier. In addition, the production of cytokines stimulates cells to form mineralized tissue (30).

Biodentine, on the other hand, acts through osteodentine mineralization by increasing the secretion of transforming growth factor-beta 1 (TGF-ϐ1) by pulp cells, along with the expression of odontoblast markers. Its setting reaction causes the formation of CH, and the consequent high pH causes an irritation that gives rise to a necrotic zone, which stimulates the migration of percussor cells that differentiate into odontoblast-like cells. Thus, there is a deposition of reactionary dentin by odontoblasts and reparative dentin through cell differentiation. That´s why biodentine also has gread sealing capacity and recommended to use for perforation repair (31,32).

When analyzing the results of Biodentine applications in long-term pulp vitality therapies, at 6, 9, 12, 24, and 36 months, all of the studies obtained considerable clinical success regardless of the therapy employed (1,4,6,13,14,18,20).

Regarding the treatment of primary teeth submitted to pulpotomy, we observed a slightly reduced success rate, with 98.90% success at three months, and 92.82% at 24 months. The success of pulp therapy in deciduous teeth corroborates the review by Jasani *et al*., 2022 (33), which shows that compared to formocresol, biodentine is a superior medicine when used in pulpotomy of deciduous teeth. Radiographically, 86.40% of success was initially observed at three months, and 89.09% at 24 months. The increase in radiographic success here is justified by the different studies analyzed in the highlighted follow-up periods.

Clinical evaluation of permanent teeth undergoing direct pulp capping therapy revealed an initial success of 98.75% at three months and a sustained success rate of 94.96% at a follow-up of 18-54 months.

Hashem *et al*. (17) reported a success rate of only 77.87% at 24-month follow-up of permanent teeth undergoing indirect pulp capping. This was the lowest clinical success rate for Biodentine obtained in the entire review. However, although the results of Hashem *et al* (17) support a lower success rate for Biodentine, this did not statistically differ from the performance of the comparison group, glass ionomer cement, which showed a 66.70% success rate.

The patients in this study manifested not only signs of reversible pulpitis, but were also recruited from an emergency department, and carried symptoms even more intense than those termed reversible pulpitis by the American Association of Endodontists, which led to less successful treatments overall (17). Thus, according to the authors, the intensity of the symptoms of reversible pulpitis also seems to be an influential factor in the success or failure of the therapy adopted.

Garrocho-Rangel *et al*. (16) support the significance of this fact, as they suggest the success rate was associated not with the use of an appropriate material and selective removal of infected dentin. Since these factors were similar in both studies. But the success rates derived from the appropriate clinical pulpal diagnosis.

One of the significant findings of this review was the divergent relationship between the clinical and radiographic success in the long-term follow-up. The results of this study point to a premature decrease in the radiographic success of Biodentine, at three months (6), with no significant modification over time; that is, the radiographic success rate is independent of time. However, the clinical data, where an approximate reduction was only observed at 12 months of follow-up, showed a linear reduction over time (4). Therefore, the importance of radiographic monitoring for treatment evaluation is emphasized, even in the absence of clinical symptoms (15).

It is also evident that success rates, in general, are dependent on several factors and can be considered subjective. Despite considerable consensus in designating a treatment as clinically successful, the same criteria do not apply to radiographic success. There is disagreement among which variables dictate success radiographically, such as internal root resorption, pulp canal obliteration, and dentin bridge formation, which are contradictory factors, thus preventing a homogeneous analysis (20). For example, dentin bridge formation, considered a radiographic success factor in several articles, proved difficult to locate due to the insufficient radiopacity of Biodentine, making it difficult to distinguish and consequently evaluate (9).

According to the results of this review, Biodentine is a reliable material for applications in vital pulp therapy treatments, maintaining adequate clinical success for deciduous and permanent teeths in long-term follow-ups. Therefore, the proven long-term effectiveness combined with the benefits of Biodentine, including a shorter setting time, lack of tooth discoloration, higher strength, and easier handling, make this material promising for clinical use.

## Figures and Tables

**Table 1:** Quality assessment analysis of the studies included in the systematic review.

Author, year	Sample Calculation	Randomization	Control Group	Blinding	Methodological details	Calibration	Total
HASHEM et al., 2019 (17)	1	1	1	0	1	1	5
ÇELIL et al., 2019 (4)	1	1	1	0	1	1	5
AWAWDEH et al., 2018 (9)	1	1	1	0	1	1	5
CARUSO et al., 2018 (15)	1	0	1	1	1	1	5
PARINYAPROM et al., 2018 (19)	1	1	1	0	1	1	5
CARTI, OZNURHAN, 2017 (6)	1	1	1	0	1	0	4
GARROCHO-RANGEL et al., 2017 (16)	1	1	1	1	1	1	6
KATGE, PATIL, 2017 (18)	1	1	1	0	1	1	5
RAJASEKHARAN et al., 2017 (20)	1	1	1	0	1	1	5
BRIZUELA et al., 2017 (14)	1	1	1	0	1	1	5
BANI et al., 2017 (13)	1	1	1	0	1	1	5
CUADROS-FERNÁNDEZ et al., 2016 (1)	1	1	1	0	1	1	5

**Table 2:** Analysis of the certainty of evidence from clinical trials included in the systematic review.

Certainty assessment							№ of patients		Effect		Certainty	Importance
№ of studies	Study design	Risk of bias	Inconsistency	Indirectness	Imprecision	Other considerations	[Biodentin]	[No comparation]	Relative(95% CI)	Absolute(95% CI)		
Clinical analysis of primary teeth submitted to pulpotomy												
6	randomised trials*	not serious	not serious	not serious	not serious	publication bias strongly suspectedvery strong association	798/843 (94.7%)		--(95.151 to 97.600)	-- per 1.000(from -- to --)	⨁⨁⨁⨁High	IMPORTANT
Clinical analysis of permanent teeth submitted to direct pulp capping												
3	randomised trials	not serious	not serious	not serious	not serious	very strong association	315/321 (98.1%)		--(95.5 to 99.0)	-- per 1.000(from -- to --)	⨁⨁⨁⨁High	IMPORTANT
Radiographic analysis of primary teeth submitted to pulpotomy												
6	randomised trials*	not serious	serious	not serious	not serious	very strong association	763/839 (90.9%)		--(87.9 to 91.8)	-- per 1.000(from -- to --)	⨁⨁⨁⨁High	IMPORTANT
Clinical analysis of primary teeth submitted to indirect pulp capping												
1	randomised trials	not serious	not serious	not serious	not serious	none	203/205 (99.0%)		--(97.624 to 99.964)	-- per 1.000(from -- to --)	⨁⨁⨁⨁High	IMPORTANT
Radiographic analysis of permanent teeth submitted to indirect pulp capping												
1	randomised trials	not serious	not serious	not serious	not serious	very strong association	41/42 (97.6%)		--(93.629 to 99.838)	-- per 1.000(from -- to --)	⨁⨁⨁⨁High	IMPORTANT

* The study by Caruso (*et al.*, 2018), is the only work included that is a retrospective study.

## Data Availability

The datasets used and/or analyzed during the current study are available from the corresponding author.

## References

[1] Cuadros-Fernández C, Rodríguez AL, Sáez-Martínez S, García-Binimelis J, Mercadé M (2016). Short-term treatment outcome of pulpotomies in primary molars using mineral trioxide aggregate and Biodentine: a randomized clinical trial. Clin Oral Investig.

[2] Saghiri MA, Asatourian A, Garcia-Godoy F, Sheibani N (2016). Effect of biomaterials on angiogenesis during vital pulp therapy. Dent Mater J.

[3] Wells C, Dulong C, McCormack S (2019). Vital pulp therapy for endodontic treatment of mature teeth: a review of clinical effectiveness, cost-effectiveness, and guidelines. CADTH.

[4] Çelik BN, Mutluay MS, Arıkan V, Sarı Ş (2019). The evaluation of MTA and Biodentine as a pulpotomy materials for carious exposures in primary teeth. Clin Oral Investig.

[5] Duarte MAH, Marciano MA, Vivan RR, Tanomaru M, Tanomaru JMG, Camilleri J (2018). Tricalcium silicate-based cements: properties and modifications. Braz Oral Res.

[6] Carti O, Oznurhan F (2017). Evaluation and comparison of mineral trioxide aggregate and biodentine in primary tooth pulpotomy: Clinical and radiographic study. Niger J Clin Pract.

[7] Sousa NB, Nunes MADC, Veloso KMM, Pereira ADFV (2014). Mineral trioxide aggregate and the use as a material for retrofilling endodontic surgery. Braz Dent J.

[8] Marciano MA, Camilleri J, Lucateli RL, Costa RM, Matsumoto MA, Duarte MAH (2019). Physical, chemical, and biological properties of white MTA with additions of AlF3. Clin Oral Investig.

[9] Awawdeh L, Al-Qudah A, Hamouri H, Chakra RJ (2018). Outcomes of vital pulp therapy using mineral trioxide aggregate or Biodentine: a prospective randomized clinical trial. J Endod.

[10] Rajasekharan S, Martens LC, Cauwels RGEC, Anthonappa RP (2018). Biodentine™ material characteristics and clinical applications: a 3 year literature review and update. Eur Arch Paediatr Dent.

[11] Coecke S, Balls M, Bowe G, Davis J, Gstraunthaler G, Hartung T (2005). Guidance on good cell culture practice. a report of the second ECVAM task force on good cell culture practice. Altern Lab Anim.

[12] Santin GC, Oliveira DSB, Galo R, Borsatto MC, Corona SAM (2014). Antimicrobial photodynamic therapy and dental plaque: a systematic review of the literature. Sci World J.

[13] Bani M, Aktaş N, Çınar Ç, Odabaş ME (2017). The clinical and radiographic success of primary molar pulpotomy using Biodentine™ and mineral trioxide aggregate: a 24-month randomized clinical trial. Pediatr Dent.

[14] Brizuela C, Ormeño A, Cabrera C, Cabezas R, Silva CI, Ramírez V (2017). Direct pulp capping with calcium hydroxide, mineral trioxide aggregate, and biodentine in permanent young teeth with caries: a randomized clinical trial. J Endod.

[15] Caruso S, Dinoi T, Marzo G, Campanella V, Giuca MR, Gatto R (2018). Clinical and radiographic evaluation of biodentine versus calcium hydroxide in primary teeth pulpotomies: a retrospective study. BMC Oral Health.

[16] Garrocho-Rangel A, Quintana-Guevara K, Vázquez-Viera R, Arvizu-Rivera JM, Flores-Reyes H, Escobar-García DM (2017). Bioactive tricalcium silicate-based dentin substitute as an indirect pulp capping material for primary teeth: a 12-month follow-up. Pediatr Dent.

[17] Hashem D, Mannocci F, Patel S, Manoharan A, Watson TF, Banerjee A (2019). Evaluation of the efficacy of calcium silicate vs. glass ionomer cement indirect pulp capping and restoration assessment criteria: a randomised controlled clinical trial-2-year results. Clin Oral Investig.

[18] Katge FA, Patil DP (2017). Comparative analysis of 2 calcium silicate-based cements (Biodentine and Mineral Trioxide Aggregate) as direct pulp-capping agent in young permanent molars: a split mouth study. J Endod.

[19] Parinyaprom N, Nirunsittirat A, Chuveera P, Lampang SN, Srisuwan T, Sastraruji T (2018). Outcomes of direct pulp capping by using either ProRoot mineral trioxide aggregate or Biodentine in permanent teeth with carious pulp exposure in 6-to 18-year-old patients: a randomized controlled trial. J Endod.

[20] Rajasekharan S, Martens LC, Vandenbulcke J, Jacquet W, Bottenberg P, Cauwels RGEC (2017). Efficacy of three different pulpotomy agents in primary molars: a randomized control trial. Int Endod J.

[21] Cengiz E, Yilmaz HG (2016). Efficacy of Erbium, Chromium-doped:Yttrium, Scandium, Gallium, and Garnet Laser Irradiation Combined with Resin-based Tricalcium Silicate and Calcium Hydroxide on Direct Pulp Capping: A Randomized Clinical Trial. J Endod.

[22] Sharma N, Malik N, Garg Y, Singh H, Garg K, Bagaria A (2019). Comparative Evaluation of Effect of Lasers and Biodentine in Dentine Regeneration: A Clinical Study. J Contemp Dent Pract.

[23] E A, Gyanendra K, Dhillon JK (2019). Comparative evaluation of clinical outcome of indirect pulp treatment with calcium hydroxide, calcium silicate and Er,Cr:YSGG laser in permanent molars. Laser Ther.

[24] Yazdanfar I, Barekatain M, Zare Jahromi M (2020). Combination effects of diode laser and resin-modified tricalcium silicate on direct pulp capping treatment of caries exposures in permanent teeth: a randomized clinical trial. Lasers Med Sci.

[25] Koubi G, Colon P, Franquin JC, Hartmann A, Richard G, Faure MO (2013). Clinical evaluation of the performance and safety of a new dentine substitute, Biodentine, in the restoration of posterior teeth - a prospective study. Clin Oral Investig.

[26] Shafaat OS, Jamil AR, Shazia AS, Maryam M (2016). A comparison of the human pulpal pain response to biodentine and mineral trioxide aggregate as pulp capping agent. Pak Oral Dent J.

[27] Hashem D, Mannocci F, Patel S, Manoharan A, Brown JE, Watson TF (2015). Clinical and radiographic assessment of the efficacy of calcium silicate indirect pulp capping: a randomized controlled clinical trial. J Dent Res.

[28] Zanini M, Hennequin M, Cousson PY (2019). Which procedures and materials could be applied for full pulpotomy in permanent mature teeth? A systematic review. Acta Odontol Scand.

[29] Krastl G, Weiger R, Ebeleseder K, Galler K (2022). Present status and future directions: Endodontic management of traumatic injuries to permanent teeth. Int Endod J.

[30] Costa DD, Mariano MMC, Muniz YS, Duplat CBS, Patrocínio DSJ, Santos JLS (2012). Mineral trioxide aggregate-A review of its composition, mechanism of action, and clinical indications. Health Magazine. com.

[31] Priyalakshmi S, Ranjan M (2014). Review on Biodentine-a bioactive dentin substitute. IOSR J Dent Med Sci.

[32] Patel M, Patel H, Kesharani P, Jani k, Shah k, Kapadia U (2023). Evaluation of Sealing Ability of MTA Flow, biodentine and Pro-root MTA to Seal the furcal perforation with and without internal matrix- an In vitro study. J Pharm Bioallied Sci.

[33] Jasani B, Musale P, Jasani B (2022). Efficacy of Biodentine versus formocresol in pulpotomy of primary teeth: a systematic review and meta-analysis. Quintessence Int.

